# *Salmonella* Fecal Shedding and Immune Responses are Dose- and Serotype- Dependent in Pigs

**DOI:** 10.1371/journal.pone.0034660

**Published:** 2012-04-16

**Authors:** Renata Ivanek, Julia Österberg, Raju Gautam, Susanna Sternberg Lewerin

**Affiliations:** 1 Department of Veterinary Integrative Biosciences, College of Veterinary Medicine and Biomedical Sciences, Texas A&M University, College Station, Texas, United States of America; 2 Department of Animal Health and Antimicrobial Strategies, Swedish National Veterinary Institute, Uppsala, Sweden; 3 Dept. of Biomedical Sciences and Veterinary Public Health, Swedish University of Agricultural Sciences, Uppsala, Sweden; Institut Pasteur, France

## Abstract

Despite the public health importance of *Salmonella* infection in pigs, little is known about the associated dynamics of fecal shedding and immunity. In this study, we investigated the transitions of pigs through the states of *Salmonella* fecal shedding and immune response post-*Salmonella* inoculation as affected by the challenge dose and serotype. Continuous-time multistate Markov models were developed using published experimental data. The model for shedding had four transient states, of which two were shedding (continuous and intermittent shedding) and two non-shedding (latency and intermittent non-shedding), and one absorbing state representing permanent cessation of shedding. The immune response model had two transient states representing responses below and above the seroconversion level. The effects of two doses [low (0.65×10^6^ CFU/pig) and high (0.65×10^9^ CFU/pig)] and four serotypes (*Salmonella* Yoruba, *Salmonella* Cubana, *Salmonella* Typhimurium, and *Salmonella* Derby) on the models' transition intensities were evaluated using a proportional intensities model. Results indicated statistically significant effects of the challenge dose and serotype on the dynamics of shedding and immune response. The time spent in the specific states was also estimated. Continuous shedding was on average 10–26 days longer, while intermittent non-shedding was 2–4 days shorter, in pigs challenged with the high compared to low dose. Interestingly, among pigs challenged with the high dose, the continuous and intermittent shedding states were on average up to 10–17 and 3–4 days longer, respectively, in pigs infected with *S.* Cubana compared to the other three serotypes. Pigs challenged with the high dose of *S.* Typhimurium or *S.* Derby seroconverted on average up to 8–11 days faster compared to the low dose. These findings highlight that *Salmonella* fecal shedding and immune response following *Salmonella* challenge are dose- and serotype-dependent and that the detection of specific *Salmonella* strains and immune responses in pigs are time-sensitive.

## Introduction

Salmonellosis is a major public health burden that contributes to the significant economic cost worldwide [1]. Pigs are an important source of salmonellosis in humans transmitted through the consumption of *Salmonella* contaminated pork products [2], [3] and through the direct contact with infected pigs [4]. Therefore, *Salmonella* detection measures are critical for efficient *Salmonella* control in pigs, that will eventually decrease *Salmonella* exposure and infection risks to humans.

Although *Salmonella* infection in pigs is frequent, it is seldom associated with clinical disease [5]. Furthermore, fecal shedding of *Salmonella* in pigs is characterized by variable durations and an intermittent shedding pattern, that is discontinuous (or alternating below and above detection level) excretion of the pathogen from the host. Asymptomatic infection and intermittent shedding pose a great problem in the detection and control of *Salmonella* in pigs. Therefore, immune response to *Salmonella* infection is often used to screen for *Salmonella* infection in pigs [6]. However, it is impossible to differentiate between the concurrent and past infections based on the serum titer. Also, the level of detectable antibodies may vary during the infection period and may be low or absent even in concurrently infected pigs. To improve detection and control of *Salmonella* in live pigs, it is critical to better understand the duration and dynamics of intermittent *Salmonella* fecal shedding and immune response post exposure and during infection, together with the factors that affect these processes.

Previous studies suggest that *Salmonella* serotype and dose affect the infection process. Specifically, it is reported that the dose of exposure affects fecal shedding [7]–[10] and immune response [8]–[10]. Differences in shedding and immune response are also reported among *Salmonella* serotypes [9]–[12]. Therefore, we hypothesize that pigs exposed to different *Salmonella* serotypes at different doses may show different patterns and dynamics of *Salmonella* fecal shedding and immune response. Such information may have important implications in aiding the early detection of *Salmonella* in pigs, leading to its more effective control at the farm level. Detailed data on infection dynamics are also essential for further work on mathematical modeling of *Salmonella* transmission and intervention strategies.

Addressing this hypothesis requires longitudinal experimental data on fecal shedding and immune response post challenge with various *Salmonella* doses and serotypes tested (such as [9], [10]) and a method that allows simultaneous analysis of multiple and reversible events. A multistate model has been used frequently in medicine to study diseases where patients may experience several events during follow-up (e.g., [13]). It is defined as a model for a stochastic process where an individual at any time occupies one of a set of multiple discrete states [14]. In the multistate model, a change of state, called a transition, is an event of interest, and the model structure specifies the states and possible transitions [14]. Compared to the Cox proportional hazards model, an alternative approach for analysis of the survival-type data, the multistate model has advantages as it offers a better understanding of the disease process, providing the hazard for movement out of one state into another (transition intensities) as affected by the covariates using a proportional intensities model, as well as the mean time spent in each state (sojourn time) [13], [15].

The objective of this study was to investigate the transition of pigs through different states of *Salmonella* fecal shedding and serological responses post *Salmonella* challenge as affected by different challenge doses and serotypes. This was accomplished by application of multistate modeling using our previously published experimental challenge data on *Salmonella* in pigs [9], [10].

## Methods

### Description of data

For the purpose of this study, the datasets from [9], [10] were combined and throughout this article we refer to the combined dataset as the “pig dataset”. Briefly, in the pig dataset 10-week old pigs were inoculated with low (0.65×10^6^ CFU) or high (0.65×10^9^ CFU) dose of one of the four serotypes of *Salmonella enterica* subsp. *enterica*: Yoruba, Cubana, Typhimurium, or Derby (hereafter abbreviated as *S.* Yoruba, *S.* Cubana, *S.* Typhimurium, and *S.* Derby, respectively). There were 8 serotype-dose combinations and 6 pigs in each serotype-dose group, resulting in a total of 48 pigs included in the dataset.

Pigs were monitored for 8 weeks for fecal excretion of the four *Salmonella* serotypes. Among pigs challenged with the high dose, 2 pigs in each group were monitored for an additional 2 weeks. Pigs were sampled once before challenge to confirm their infection-free status. Furthermore, they were sampled every day from the day of challenge (day 0) to day 5, and every 1–4 days thereafter. A total of 1,215 fecal samples were collected during the study. *Salmonella* detection was based on microbial culture with modified semi-solid Rappaport Vassiliadis (MSRV) enrichment, reported to have sensitivity and specificity of detecting *Salmonella* in pig fecal samples of 95% and 100%, respectively, and detection limit of 10 CFU/25 gram sample [16]. The results were in the form of presence or absence of the particular *Salmonella* serotype in feces of a pig on a given day.

Blood samples were collected once a week for 8 weeks starting from day 0 of the experiment. Among pigs challenged with the high dose, 2 pigs were selected from each serotype group and their blood samples were collected once a week for the additional 2 weeks. A total of 446 serum samples were collected during the study period. To detect serum antibodies, commercial ELISA tests were used: “Herdcheck Swine *Salmonella*” from IDEXX Laboratories for *S.* Derby and *S.* Cubana (based on LPS-antigens from the *Salmonella* serogroups B, C1, and D) and “Svanovir” from Svanova Biotech for *S.* Typhimurium (based on antigens from *S.* Typhimurium and *S.* Choleraesuis (O antigens 1, 4, 5, 6, 7, and 12)). An in-house ELISA test was used to detect serum antibodies to *S.* Yoruba (O antigen 16). The results of ELISA tests were in the form of the level of antibodies detected in a pig on a given day.

### Multistate models

The longitudinal nature of the pig dataset provides information on the presence or absence of *Salmonella* in feces and the level of immune response in pigs' sera during infection. Furthermore, the dataset provides information on the patterns of shedding and immune response over the course of infection which could be translated into allowed states and transitions in the corresponding multistate models. Specifically, it is easy to comprehend the difference in the meaning of a positive or negative test result depending on the elapsed time from the day of challenge that a particular sample of feces or blood was collected and examined. For example, absence of *Salmonella* from feces has a different meaning on the first day after challenge, in the middle of *Salmonella* infection and at the end of the infection, possibly indicating latency, intermittent non-shedding and recovery, respectively (which we define in the next paragraph).

#### Fecal shedding multistate model

The semiquantitative data on the level of *Salmonella* excretion during infection reported in [9], [10] indicate that shedding levels, although overall slightly decreasing towards the end of infection, were highly variable throughout the infection. Moreover, based on the shedding levels observed during infection in the individual pigs, there were blocks of sampling times when the pathogen was not detected scattered in-between the blocks of shedding with variable levels of *Salmonella*. Thus, the observed intermittent shedding cannot be adequately explained by considering a monotonic decline in the shedding level following challenge. Considering sensitivity, specificity and detection limit of MSRV, it is reasonable to assume that the observed culture results represent the true status of animals with a reasonable degree of accuracy. Thus, combining the information on the culture result and its timing from the day of challenge, several facts were observed for fecal shedding in the pig dataset. *Salmonella* was never detected post challenge in feces of some pigs. Not all pigs started shedding immediately post-challenge. When pigs finally started shedding post-challenge, they may shed continually (i.e., without detected ceasing) for a variable period of time. After this period, some pigs were never found shedding again, while shedding could be detected on and off for variable short periods of time in other pigs. In the dataset, some of these latter pigs completely stopped shedding after some time while others continued this intermittent pattern of shedding until the study was terminated. To reflect these observations, the pig dataset was interpreted and states were defined as follows:

- 1 =  latency: negative sample on the day of and immediately after challenge;
- 2 =  continuous shedding state: an uninterrupted sequence of positive samples after challenge;
- 3 =  intermittent non-shedding state: negative samples following state 2 or state 4 if they were followed by positives before the end of the experiment;
- 4 =  intermittent shedding state: positive samples following state 3;
- 5 =  recovery: negative samples following state 1, 2, or 4 if there were at least 5 consecutive negatives during at least a 14-day period. Recovery following shedding states (states 2 and 4) represents clearance of *Salmonella* from feces. Recovery following latency (state 1) indicates that the pig was never found to shed *Salmonella* in feces, suggesting that it did not become infected after exposure (i.e., the pig was in the latent state on the day of challenge and considered recovered thereafter). These conditions for recovery were chosen to increase specificity in classifying pigs as recovered and to prevent false classification of positive pigs as recovered;
- 99 =  censored state: if a sample was negative but conditions for state 5 were not met, it is unknown whether *Salmonella* was not detected because the pig has recovered from infection or because it has been in the temporary non-shedding state. In other words, censoring means that the observed state is known only to be one of a particular set of states (i.e., 3 and 5). Note that in contrast to the usual terminology of survival analysis, here it is the state which is considered to be censored, rather than the event time [17];
- NA =  sample not collected/examined.

**Figure 1:**
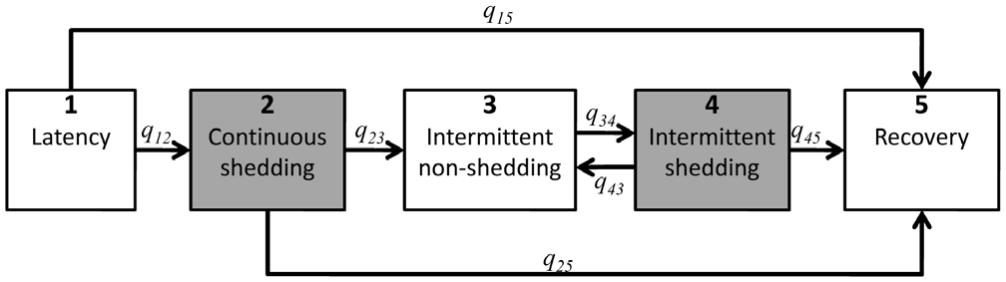
A multistate model describing transition of pigs through the states of shedding following challenge. At time t an individual is in state *i*. Its 5 states are labeled 1, 2, 3, 4, and 5, denoting: 1-latency: the individual has been challenged but has not yet started (or will never start) shedding; 2-continuous shedding state; 3-intermittent non-shedding state; 4-intermittent shedding state; and 5-recovery: clearance of *Salmonella* from feces. Gray shaded compartments denote the pigs that excrete detectable levels of *Salmonella* in feces.

The considered multistate fecal shedding model is shown in Figure 1. It includes four transient states (*i* = 1, 2, 3, and 4) and one absorbing state (*i* = 5). The arrows in Figure 1 (labeled with *q_ij_*) show which instantaneous transitions are possible between states.

#### Immune response multistate model

The level of antibodies detected in a pig's serum on a given day was dichotomized using a cut-off so that the pig could be classified as having an immune response below or above the seroconversion level on a given day. The individual variation in the humoral immune response rules out the possibility to perfectly divide animals into these two groups. In the commercial ELISA kits, the validation of the cut-off has been performed by testing a large number of pig reference sera. In the [9] study, the in-house ELISA was used to detect antibodies to *S.* Yoruba, and the cut-off was calculated by using the mean of the samples collected pre-inoculation, i.e., when pigs were *Salmonella*-negative, and by adding two standard deviations. According to this cut-off, 2 pigs would be unrealistically classified as seroconverted already on day 0 of the experiment. To correct for that, for the purpose of this study, a “custom” cut-off for the in-house ELISA was defined as 1.5× of the highest antibody level detected on day 0. This was done to raise the specificity for detection of antibodies to *S.* Yoruba in order to avoid possibly false positive classifications in the model. For consistency in detecting antibodies to other *Salmonella* serotypes in the study, we applied the same custom cut-off in classification of the other ELISA tests results. However, because the use of a custom cut-off might have reduced the specificity of the commercial ELISA kits, we also ran the analysis using the data where the manufacturer recommended cut-offs were used to classify *S.* Cubana, *S.* Derby, and *S.* Typhimurium, while the custom cut-off was used to classify *S.* Yoruba ELISA data.

**Figure 2:**
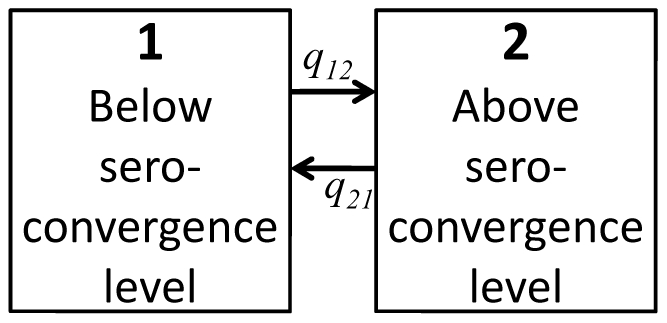
A multistate model describing transition of pigs through the states of immune response following challenge. At time t an individual is in state *i*. Its 2 states are labeled 1 and 2, denoting immune response below or above the seroconversion level, respectively.

Combining the information on the serological test result and its timing from the day of challenge, the following was observed for pigs' immune responses to *Salmonella* challenge. Some pigs did not seroconvert post challenge. In other pigs, the antibody level rose post-challenge at variable rates. In some pigs that seroconverted, the detected antibody level temporarily fell below the cut-off. Accordingly, negative and positive blood samples were coded with 1 and 2 indicating immune response below and above the seroconversion level, respectively. The considered multistate model for immune response (Figure 2) includes two transient states that represent a pig's dichotomized immune response during infection, i.e., immune response below and above the seroconversion level (*i* = 1 and 2). The instantaneous transitions from state to state in the model shown in Figure 2 are represented by the intensities *q_ij_*.

#### Multistate models: analyses, assumptions, selection and validation

The reader is directed elsewhere to obtain information about the theory underlying the multistate models (e.g., [13], [14], [17], [18]). In the current study, all analyses and modeling were done in R [19], using the msm package [20]; R codes are available on request from the first author.

In the pig dataset, the sampling times were assumed to be non-informative, observation times were fixed, and the transition times were not exact. The effect of serotype and dose on the dynamics of fecal shedding and immune response was quantified by examining their effect on (i) the hazard ratios, (ii) ratios of transition intensities when more than one transition out of a state is possible, and (iii) the time spent in each state (the sojourn and total time).

In specifying the multistate models, the following assumptions were made: (1) the transition times from each state are independent of the history of the process prior to entry to that state (the Markov assumption), (2) transition intensities are homogeneous across the pig population, and (3) transition intensities are homogeneous through time. Assumption (1) is inherent to many multistate models; it could not be tested due to absence of data on the exact transition times [21]. Assumption (2) was tested by including covariates in the model through a model selection process and comparing it with the model without covariates (H_0_). Model selection involved forward selection of covariates associated significantly with the transition intensities. The best model was selected based on the Likelihood ratios (LR) and Akaike's Information Criterion (AIC). In such a saturated model the effect of the covariate differs in each of the allowed transitions (i.e., 7 and 2 transitions in Figures 1 and 2, respectively). So, for example, in the fecal shedding model with just one covariate, there will be a total of 14 parameters, 7 baseline transition intensities, and 7 different regression coefficients. We compared the saturated model with models with a reduced number of parameters achieved by biologically meaningful constraining of the effect of a covariate to be equal for certain transition intensities. Then, LR and the associated p-value at the 5% level were used to select the most parsimonious representation of the association between each covariate and the transition intensities [13]. Further, we tested the existence of interactions among covariates in the model. Assumption (3) was assessed by fitting a model that allows parameters to be stepwise constant through time using a piecewise-constant function [20] and comparing the fit of this model to the model with homogeneous transition intensities [18].

In the msm package, the likelihood function is maximized by numerical methods, which needs a set of initial values to start searching for the maximum. A function “crudeinits.msm” was used to obtain initial values for the specified multistate models using the observed number of transitions from state *i* to state *j* and the observed total time spent in state *i* (as in [20]). This approach assumes that there are not many changes of states in between the observation times.

Ideal data for model validation would be from a similar longitudinal study of fecal shedding and immune responses obtained from an independent experiment of the same challenge doses and serotypes. However, to our best knowledge, such data do not exist. Therefore, we tested internal validity of the models by comparing the observed and model-predicted (mean and 95% confidence intervals [CI]) prevalences of pigs in different states. CI were estimated by simulating 1,000 random vectors from the asymptotic multivariate normal distribution implied by the maximum likelihood estimates (and covariance matrix) of the log transition intensities and covariate effects, then calculating the expected prevalences for each replicate [22]. Wherever possible, we also compared model outputs with the literature to assess the generalizability of our findings.

## Results

### Fecal shedding model

In the pig dataset, *Salmonella* was detected in 532 out of 1,215 fecal samples. Of these positive samples, 344 denoted the presence of a pig in the continuous shedding state and 188 in the intermittent shedding state. There were 683 negative samples, of which 56, 202, and 371 were classified into the latent, intermittent non-shedding and recovered states, respectively, while 54 samples were censored (i.e., the pig was considered to be either in the intermittent non-shedding state, or the recovered state). There were 304 observations each of *S.* Cubana, *S.* Typhimurium, and *S.* Derby, and 303 of *S.* Yoruba. For the low and high challenge doses, there were 575 and 640 observations, respectively. Initial values of parameters in the shedding model were *q_12_* = 0.75, *q_15_* = 0.11, *q_23_* = 0.06, *q_25_* = 0.01, *q_3_*_4_ = 0.16, *q_43_* = 0.10, and *q_45_* = 0.03.

**Figure 3:**
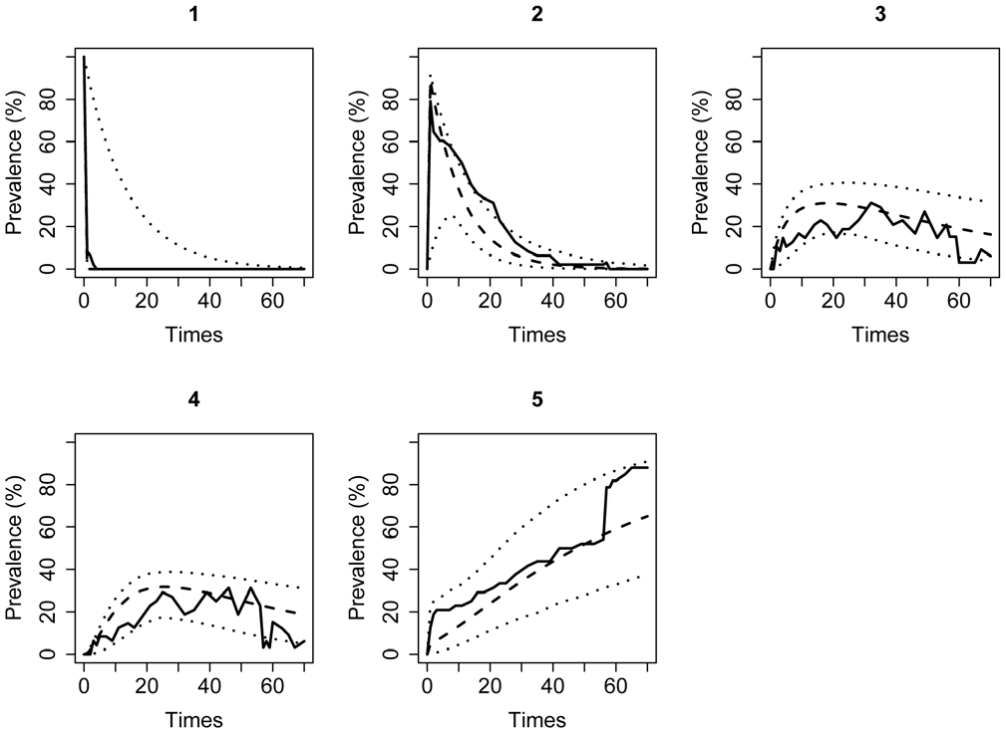
Observed versus expected prevalence of pigs in the states of the fecal shedding model. The 5 states are labeled 1, 2, 3, 4, and 5, denoting: 1-latency; 2-continuous shedding; 3-intermittent non-shedding state; 4-intermittent shedding state; and 5-recovery. Full line indicates the observed, while dashed and dotted lines denote mean and 95% CI of the expected prevalence.

A single-covariate multistate model was used to assess the individual effects of covariates on the transition intensities in the multistate models and to find the most parsimonious representation of the covariate using LR and AIC tests. The low challenge dose and *S.* Cubana were used as the reference groups. Compared to the H_0_ model without covariates (AIC = 1021.2), these single-covariate analyses indicated a statistically significant association between the model transition intensities and serotype with constraints *q_15_* = *q_25_* = *q_45_* = −*q_23_* = *−q_43_*, meaning an equal effect of serotype on all recovery transitions, i.e., *q_15_*, *q_25_*, and *q_45_*, and equal effect but in opposite direction on the transitions to the intermittent non-shedding state, i.e., *q_23_* and *q_43_* (LR = 21.6, degrees of freedom (df) = 16-7 = 9, χ^2^ p-value = 0.01; AIC = 1017.6). The dose was found to be associated with transition intensities in the saturated form (LR = 61.6, df = 14-7 = 7, χ^2^ p-value<10^−10^; AIC = 973.6). Multivariate modeling results further indicated that the best model for fecal shedding dynamics includes both the challenge serotype (in the above described constrained form) and dose (in the saturated form). A comparison of the observed and model-predicted prevalences with 95% CI for this model indicated a reasonably good fit to the data used for its development and so supported the internal validity of the model (Figure 3). Testing for evidence of interactions and time heterogeneity in the model was not possible due to a non-positive definite, and hence not invertible, Hessian matrixes. When a Hessian is not invertible, no computational trick can make it invertible, given the model and data chosen [23].

From the final fecal shedding model, we estimated hazard ratios (HR) and the corresponding 95% CI. Compared to pigs challenged with the low dose of *Salmonella*, pigs challenged with the high dose had a statistically significantly reduced risk of transition from the continuous shedding state to the intermittent non-shedding state (HR = 0.3, 95% CI: 0.2, 0.6), from the continuous shedding state to recovery (HR = 0.1, 95% CI: 0.01, 0.4), and from the intermittent shedding state to recovery (HR = 0.1, 95% CI: 0.02, 0.6). That means that pigs challenged with the high dose tend to spend more time shedding both as part of the continuous and intermittent shedding states compared to those challenged with the low dose. Compared to pigs infected with *S.* Cubana, pigs infected with *S.* Typhimurium or *S.* Derby had significantly reduced risks of recovery (from latency, continuous shedding, and intermittent shedding states), both having HR = 0.4 (95% CI: 0.2, 0.8). On the other hand, the risk of transition to intermittent non-shedding either after the continuous or intermittent shedding, was significantly higher in pigs infected with *S.* Typhimurium (HR = 2.6, 95% CI: 1.2, 5.5) or *S.* Derby (HR = 2.5, 95% CI: 1.3, 4.9), compared to *S.* Cubana. Taken together, this means that pigs challenged with *S.* Cubana are less likely to become infected compared to *S.* Typhimurium and *S.* Derby. However, when they do become infected they will tend to go through longer continuous and intermittent shedding states after which they will be more likely to recover than *S.* Typhimurium and *S.* Derby (which will tend to transition between the intermittent shedding and non-shedding states more often with a short period of occupancy of these states at each visit). Therefore, the total duration of *S.* Cubana infection will likely be shorter compared to *S.* Typhimurium and *S.* Derby. There was no evidence of the statistically significant effect of dose and serotype on the other model transitions. Likewise, there was no evidence of any difference between *S.* Cubana and *S.* Yoruba.

Table 1 shows estimated ratios of two entries of the transition intensity matrix at a given set of covariate levels for the fecal shedding model with the dose and serotype considered, together with approximate standard errors estimated using the delta method [20]. These results indicate that challenge dose and serotype have strong effects on the probability that a pig will exhibit a particular pattern of shedding denoted by the particular sequence and timing of visited states. That, together with the conclusions from the estimated HRs, was corroborated with the estimated mean sojourn times in a single stay (i.e., times spent in a state before leaving to another state) and the expected total length of stay in each state (Table 2). For example, continuous shedding was on average 10 days longer in pigs challenged with the high compared to low dose of *S.* Typhimurium. Thus, it is evident from Table 2 that the dose and serotype exert a pronounced effect on the duration of stay in the continuous shedding and the intermittent non-shedding states. The sojourn and expected total times in states visited only once (in our model these were latency and continuous shedding) could be the same. In our model, however, for a few covariate combinations, the estimated mean sojourn time of continuous shedding was greater than the expected total time for the state because the mean sojourn time in a state is conditional on entering the state, whereas the expected total time is a forecast for a newly exposed individual who may recover before entering the continuous shedding state. The expected total times in the intermittent shedding and non-shedding states are longer than the estimated mean sojourn times due to multiple stays in these states during infection. The expected total lengths of stays that are longer than the duration of the study should be used with caution because the study terminated before some animals recovered.

**Table 1:** Ratios of transition intensities out of a state in the fecal shedding model^1^.

Transitions^2^	dose,serotype^3^	mean	SE^4^
4→3 vs. 4→5	6,C	0.3	0.2
	6,Y	0.9	0.5
	6,D	1.8	0.9
	6,T	2.2	1.2
4→3 vs. 4→5	9,C	7.3	6.8
	9,Y	20.7	19.1
	9,D	38.7	35.7
	9,T	49.0	45.3
2→3 vs. 2→5	6,C	1.1	0.8
	6,Y	3.0	2.2
	6,D	5.7	4.0
	6,T	7.2	5.1
2→3 vs. 2→5	9,C	5.1	3.8
	9,Y	14.3	10.7
	9,D	26.7	20.1
	9,T	33.9	25.4
1→2 vs. 1→5	6,C	1.4	1.5
	6,Y	6.0	4.1
	6,D	1.6	1.1
	6,T	17.5	18.7
1→2 vs. 1→5	9,C	53.7	114.1
	9,Y	227.6	490.3
	9,D	61.3	129.8
	9,T	666.0	1523.4

1 Reference groups: low dose and *S.* Cubana.

2 Notation i→j denotes transition from state *i* to *j* with intensity *q_ij_*. The 5 states are labeled 1, 2, 3, 4 and 5, denoting: 1-latency; 2-continuous shedding; 3-intermittent non-shedding state; 4-intermittent shedding state; and 5-recovery.

3 Notations 6, 9, C, Y, D, and T denote the low dose, high dose, *S.* Cubana, *S.* Yoruba, *S.* Derby and *S.* Typhimurium, respectively.

4 SE standard errors of the mean ratios of transition intensities.

**Table 2:** Mean sojourn and the expected total times in days for transient states of the fecal shedding model^1^.

dose,serotype:state^2^	sojourn time (SE^3^)	total time
6,C:1	0.7 (0.4)	0.7
6,T:1	0.2 (0.4)	0.2
6,D:1	1.5 (0.6)	1.5
6,Y:1	0.4 (0.4)	0.4
6,C:2	5.7 (2.4)	3.3
6,T:2	3.7 (2.4)	3.5
6,D:2	4 (1.4)	2.5
6,Y:2	4.9 (2.4)	4.2
6,C:3	6.4 (3.3)	2.6
6,T:3	6.7 (3.3)	17.8
6,D:3	4.8 (1.2)	6.9
6,Y:3	9.2 (3.3)	11.5
6,C:4	4.1 (1.6)	1.6
6,T:4	4.3 (1.6)	11.6
6,D:4	4.5 (1.4)	6.5
6,Y:4	4.7 (1.6)	5.8
9,C:1	0.1 (0.4)	0.1
9,T:1	0 (0.4)	0.0
9,D:1	0.1 (0.5)	0.1
9,Y:1	0 (0.4)	0.0
9,C:2	31.1 (2.4)	30.6
9,T:2	14 (2.4)	14.0
9,D:2	15.6 (4.1)	15.4
9,Y:2	20.8 (2.4)	20.7
9,C:3	3.8 (3.3)	25.9
9,T:3	3.9 (3.3)	191.2
9,D:3	2.8 (0.8)	106.3
9,Y:3	5.5 (3.3)	110.1
9,C:4	7 (1.6)	47.6
9,T:4	3 (1.6)	145.7
9,D:4	3.4 (1)	126.6
9,Y:4	4.5 (1.6)	90.8

1 Reference groups: low dose and *S.* Cubana.

2 Notations 6, 9, C, Y, D, T, 1, 2, 3, and 4 denote the low dose, high dose, *S.* Cubana, *S.* Yoruba, *S.* Derby, *S.* Typhimurium, 1-latency, 2-continuous shedding, 3-intermittent non-shedding state, and 4-intermittent shedding state, respectively.

3 SE standard errors of the mean sojourn times.

To test the generalizability of our findings, we compared the model outputs with the available literature. In their experimental inoculation studies with a high dose (4.4×10^9^) of *S.* Typhimurium [24], observed that all pigs excreted *Salmonella* continuously until day 10 post-inoculation and that shedding became intermittent thereafter. The period during which all pigs shed *Salmonella* can be regarded as the minimum duration for which all pigs remain in the continuous shedding state. Thus, the finding by [24] is in close agreement with our estimated duration of continuous shedding for pigs inoculated with the high dose of *S.* Typhimurium (mean 14 days, 95% CI: 6, 32 days). Moreover, the [24] study confirms the existence of the continuous state and the intermittent shedding and non-shedding states; i.e., it validates the general structure of the developed multistate model. In another experimental study where pigs were inoculated orally with 10^8^ CFU of *S.* Typhimurium and tested for fecal shedding every 1 or 2 weeks for 108 days, shedding was detected up to 92 days post-inoculation [11], indicating that a long total duration of infection, longer than the duration of the follow-up in the experimental studies used here [9], [10], may occur.

### Immune response model

Of 446 serum samples in the pig dataset, using the custom cut-off, 196 and 250 samples were classified as below and above the seroconversion level, respectively. In the corresponding multistate model, the initial values were *q_12_* = 0.03, *q_21_* = 0.01. When the manufacturer recommended cut-off was used for all but *S.* Yoruba (for which we used the custom cut-off), the number of samples classified below and above the seroconversion level was 273 and 173, respectively, and in the corresponding multistate model one of the initial values was slightly different (*q_12_* = 0.02).

In the analysis of the immune response multistate model, the low challenge dose and *S.* Yoruba were used as the reference groups. The results described below pertain to the model that used ELISA data dichotomized using the custom cut-offs except when it is explicitly stated that the manufacturer recommended cut-offs were used for the commercial ELISA kits.

The single-covariate analyses indicated a statistically significant association between model transition intensities and each of the covariates, with both covariates being best represented in the reduced form such that the covariate effect is in the opposite direction between the progression and regression transitions (*q_12_* = −*q_21_*). In other words, each of the covariates showed equal but opposite effects on the progression to seroconversion and regression to the antibody level below the seroconversion level. From comparison with the model without covariates (H_0_; AIC = 285.2), statistics were as follows for the serotype: LR = 51.0, df = 5-2 = 1, χ^2^ p-value <10^−10^; AIC = 240.2, and for dose: LR = 6.4, df = 3-2 = 1, χ^2^ p-value  = 0.01; AIC = 280.8. The results of multivariate modeling indicated that the best model for immune response post-challenge has both the dose and serotype included in the above described constrained form (*q_12_* = −*q_21_*). There was no evidence of interaction between the two considered covariates and time heterogeneity in the models.

HRs estimated for this model indicated that pigs infected with the high dose had a statistically significantly higher risk of seroconverting (HR = 3.6, 95% CI: 1.9, 7.0) and a lower risk of transitioning back to the “below seroconversion level” state (HR = 0.3, 95% CI: 0.1, 0.5) than those challenged with the low dose. In other words, we can expect that pigs challenged with the high dose will seroconvert faster and remain above the serocoversion level longer. The effect of serotype on the risk of seroconversion was even more pronounced; compared to *S.* Yoruba, *S.* Typhimurium, *S.* Derby, and *S.* Cubana had HR = 16.4 (95% CI: 5.7, 47.1), HR = 18.1 (95% CI: 6.5, 50.0), and HR = 4.5 (95% CI: 1.7, 11.9), respectively. Likewise, *S.* Typhimurium, *S.* Derby, and *S.* Cubana had HR = 0.1 (95% CI: 0.02, 0.18), HR = 0.1 (95% CI: 0.02, 0.16), and HR = 0.3 (95% CI: 0.2,0.5), respectively, for returning to the “below seroconversion level” state. In other words, *S.* Typhimurium and *S.* Derby, and to a lower extent *S.* Cubana, are expected to seroconvert much faster and remain above the seroconversion level much longer than *S.* Yoruba.

To evaluate the effect of using the custom cut-off for all *Salmonella* serotypes, we also ran the analyses using the available manufacturer recommended cut-offs for the commercial ELISA kits and the custom cut-off for the in-house ELISA (used for *S.* Yoruba). According to the manufacturer recommended cut-off, none of the 12 pigs challenged with *S.* Cubana seroconverted post-challenge. Thus, *S.* Cubana data were excluded from this analysis. The covariates and constraints in the best model were identical to the one developed for the model using custom cut-offs. The estimated HRs indicated similar albeit weaker effects of covariates. For high dose vs. low dose, seroconverting and transition to the “below seroconversion level” state had HR = 2.6 (95% CI: 1.3, 5.1) and HR = 0.4 (95% CI: 0.2, 0.8), respectively. Compared to *S.* Yoruba, the other two strains, *S.* Typhimurium and *S.* Derby, had respectively HR = 7.2 (95% CI: 2.9, 18.1) and HR = 7.7 (95% CI: 3.2, 18.7) for seroconverting, and respectively HR = 0.1 (95% CI: 0.1, 0.4) and HR = 0.1 (95% CI: 0.1, 0.3) for transitioning back to the “below seroconversion level” state.

Table 3 shows estimated sojourn times for the models using custom and manufacturer cut-offs, which support conclusions from the estimated HRs. However, it should be noted that all estimates longer than the study period should be taken with caution. That is because time to seroconversion for pigs challenged with the low dose of *S.* Cubana and *S.* Yoruba could not be accurately estimated because some pigs never seroconverted. Similarly, the times spent above the seroconversion level could not be accurately estimated because the experiment was not run for a long enough time to capture waning of immunity in pigs.

**Table 3:** Sojourn times in days in states of the immune response model^1^.

	custom cut-offs		manufacturer cut-offs^2^	
dose,serotype:state^3^	mean	SE^4^	mean	SE
6,Y:1	198.3	99.1	125.1	57.9
6,C:1	44.2	15.9	NA^5^	NA
6,D:1	11.0	99.1	16.3	5.7
6,T:1	12.1	99.1	17.4	57.9
6,Y:2	6.9	3.9	7.2	3.5
6,C:2	30.8	14.8	NA	NA
6,D:2	123.4	3.9	55.4	23.3
6,T:2	112.6	3.9	52.0	3.5
9,Y:1	55.0	99.1	48.7	57.9
9,C:1	12.2	3.8	NA	NA
9,D:1	3.1	99.1	6.3	1.9
9,T:1	3.4	99.1	6.8	57.9
9,Y:2	24.8	3.9	18.5	3.5
9,C:2	111.3	47.3	NA	NA
9,D:2	445.2	3.9	142.3	55.3
9,T:2	406.3	3.9	133.5	3.5

1 Reference groups: low dose and *S.* Yoruba.

2 Manufacturer cut-offs used for all serotypes except for *S.* Yoruba.

3 Notation 6, 9, C, Y, D, T, 1 and 2 denote the low dose, high dose, *S.* Cubana, *S.* Yoruba, *S.* Derby and *S.* Typhimurium, below seroconversion level state and above seroconversion level state, respectively.

4 SE standard errors of the mean sojourn times.

5 NA = not estimated.

**Figure 4:**
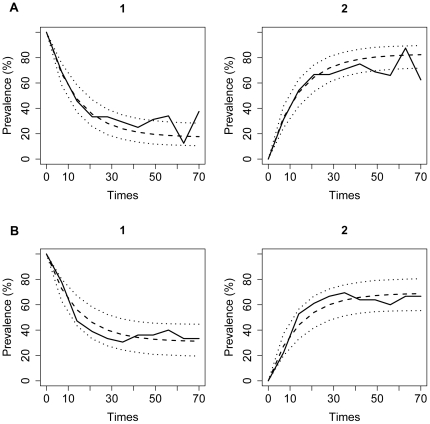
Observed versus expected prevalence of pigs in the states of the immune response model. Shown are prevalences for the models using the custom cut-offs (panel A) and manufacturer based cut-offs (for all serotypes except *S.* Yoruba; panel B). The states are labeled 1 and 2, denoting an immune response below and above the seroconversion level, respectively. Full line indicates the observed, while dashed and dotted lines denote mean and 95% CI of the expected prevalence.

Both custom and manufacturer cut-off based models showed good internal validity (Figure 4). To assess the generalizability of our findings, an external validation was performed based on available reports. In experimentally inoculated pigs with a high dose of *S.* Typhimurium, seroconversion has been detected as early as 16 days post-inoculation with the majority of pigs, with 94% (15 of 16) seroconverting between the third and fifth week (days 22 and 39) post-inoculation [24]. Similarly, pigs experimentally infected with 10^8^ of *S.* Typhimurium started seroconverting at day 7 post-inoculation with the majority of pigs seroconverting by day 22 [11]. At least 50% of pigs inoculated with 7.4×10^7^ or 3.2×10^9^ and tracer pigs housed with them seroconverted within 1–3 weeks post-inoculation [25]. These estimates overlap with the time to seroconversion in pigs challenged with the high dose of *S.* Typhimurium (7 days, 95% CI: 3, 17 days) estimated using the manufacturer recommended cut-offs for the commercial ELISA kits.

## Discussion

In this study, we used a multistate Markov modeling approach to evaluate the effect of *Salmonella* challenge dose (high v. low) and four serotypes (*S.* Yoruba, *S.* Cubana, *S.* Typhimurium, or *S.* Derby) on the dynamics of *Salmonella* fecal shedding and immune response post-challenge. We found strong evidence that the patterns and dynamics of fecal shedding and immune response are significantly and meaningfully affected by both the serotype and dose of exposure. Below we discuss these findings in the context of existing research needs and knowledge, and we elaborate on the associated implications and limitations.

Fecal shedding represents an important mechanism of spreading *Salmonella* into the environment that can eventually be transmitted to other animals and humans. Of particular concern is that *Salmonella* and many other infectious agents, such as enterohemorrhagic *Escherichia coli* and *Listeria monocytogenes* [26], [27], are characterized by intermittent shedding. Intermittent shedding is a challenge to medical and veterinary specialists because it hinders detection of infected individuals. Also, intermittent shedding certainly has an effect on the transmission of the pathogen through a population. For example, on the days when an individual is not excreting *Salmonella* in feces, the individual is not infectious to other members of the population. On the other hand, such infected individuals are not detected by fecal culture. It is therefore critical to understand the ecology and selective pressures governing intermittent fecal shedding so that intermittently-shed pathogens can be controlled more effectively. This highlights the value of the results of our study discussed below.

The analyses of the *Salmonella* fecal shedding multistate model performed in this study indicated that pigs challenged with the high dose of 10^9^ CFU of *Salmonella* on average started to shed *Salmonella* in feces up to 1.5 day sooner and, depending on the serotype, had 10–26 days longer shedding during the continuous shedding state compared to those challenged with the low dose (10^6^ CFU) of *Salmonella*. Also, intermittent non-shedding was on average 2–4 days shorter in pigs challenged with the high compared to low dose. Therefore, reduction of the dose of exposure (e.g., through cleaning and other biosecurity measures) would shorten the periods of shedding and extend the periods of intermittent non-shedding and would, consequently, decrease the spread of the infection through the population.

Our study also indicated that pigs infected with *S.* Cubana had continuous and intermittent shedding states on average up to 10–17 and 3–4 days longer, respectively, than the other three evaluated serotypes, of which *S.* Yoruba had shedding times most similar to *S.* Cubana. The similarity between *S.* Yoruba and *S.* Cubana was also supported by the lack of statistically significantly different HRs comparing their model transition intensities. Our results also indicated that compared to *S.* Yoruba and *S.* Cubana, pigs infected with *S.* Typhimurium and *S.* Derby are far more likely to enter the intermittent non-shedding state following the continuous or intermittent shedding states than to recover from these states. Overall, these findings suggest that pigs infected with *S.* Typhimurium and *S.* Derby tend to shed *Salmonella* in shorter blocks of time (both as part of the continuous and intermittent shedding states) compared to *S.* Yoruba and *S.* Cubana. However, *S.* Typhimurium and *S.* Derby are more likely to (re-)enter the intermittent non-shedding state. Consequently, they showed a tendency for longer overall duration of host infection with similar or longer total duration of fecal shedding (i.e., the sum of the continuous and all intermittent shedding episodes) compared to *S.* Yoruba and *S.* Cubana (Table 2). This trend makes sense as it may be explained by the difference in invasiveness between the more invasive *S.* Typhimurium and *S.* Derby (which are considered the classical pig serotypes) and the less invasive *S.* Yoruba and *S.* Cubana (which are mostly associated with contaminated feed). The challenge in controlling such infections is that for the invasive classical pig serotypes, ceasing or reducing pathogen replication and consequently shedding hampers their detection while seemingly it also causes the pathogen to reside in the host longer, which extends the period of time a pig is infected and thus capable of transmitting the pathogen to another host. The serotypes associated with contaminated feed are also of concern because through their feed-borne spread, many herds may become infected in a short time, and this route of transmission can therefore be regarded as a major threat to the control of *Salmonella* in pigs. Moreover, if there is no control of *Salmonella* in feed, pigs may be continuously exposed via feed, thereby producing a different shedding pattern than with only a single exposure. Indeed, the high significance of commercial feed as a potential vehicle of *Salmonella* transmission has been reported in the USA [28]. Also, *S.* Cubana has been isolated from routine environmental samples from feed mills in Sweden [29], [30], and it infected pigs in 31 herds in a feed-borne outbreak in Sweden in 2003 [31]. When *S.* Yoruba was detected in a Swedish specific pathogen-free pig herd, feed was concluded to be the most likely source [32]. The serotypes associated with contaminated feed are of concern to human health as well. In the USA, two outbreaks of human infection with *S.* Cubana were reported in 1998, affecting 34 and 14 people [33]. Because of this and similar incidents, all serotypes of *Salmonella enterica* are considered a potential threat to human health [1].

The findings of this study are obviously dependent on the correct classification of pigs into one of the possible shedding and non-shedding states, the validity of the structure of the considered multistate model for fecal shedding (i.e., the considered states and allowed transitions among states), and the underlying assumptions. With respect to a possible misclassification bias, a [16] study indicated that the MSRV method used to detect *Salmonella* in fecal samples has good diagnostic performance (sensitivity and specificity of 95% and 100%, respectively), and its detection limit is around 10 CFU/25 g feces. The number of false negatives should thus be fairly low, while there should be no false positives due to the test. The semiquantitative results for the level of *Salmonella* in feces of individual pigs in the experimental studies used here [9], [10] indicated that the blocks of non-shedding were distributed among the blocks of shedding of variable levels of *Salmonella*. This, together with the MSRV's diagnostic performance and the detection limit, supports the finding that the intermittent non-shedding state truly occurs as opposed to representing blocks of false negative classifications. Also, it is unlikely that we would observe just by chance the inverse relationship between the strain-specific duration of a one-time stay in the fecal shedding states and the total duration of infection and shedding. Therefore, misclassification bias does not seem to be of high concern in this study. Any misclassification would be rather in the form of assuming non-shedding instead of low-shedding. The conclusions and the biological relevance would remain the same, as low-shedding (below the detection limit) would have more or less the same effect on detection and spread as non-shedding. Nevertheless, a formal assessment of this question could be done through an extension of the developed model into a Hidden Markov model in a future study.

While intermittent shedding is widely known to occur during infection with *Salmonella* and many other pathogens, it has rarely been specifically considered in models of the within-host or among host-infection dynamics. The [34] study modeled recurrence of infectiousness through fecal shedding by inclusion of the carrier state into the model of the *S.* Typhimurium transmission within a pig herd. Similarly, the [35] study modeled the transition of cattle between the states of shedding and non-shedding of *L. monocytogenes* in feces. However, to our knowledge, the study described here is unique in the way it explicitly separated the shedder animals into those that are in the continuous and intermittent shedding states, which has important implications for infection detection and transmission, as already discussed. Because samples were not collected daily, it is possible that some pigs were negative even before the first negative sample was obtained. Therefore, the duration of continuous shedding might have been overestimated. However, given the fairly similar frequency of sample collection before and after the first negative sample was collected, and the favorable validation with [24], it is highly unlikely that the continuous shedding state is just an illusion and that the associated estimates are just artifacts.

Our model of the within-host dynamics of fecal shedding assumes a single challenge at day 0. It completely ignores the possibility of pig-to-pig transmission of *Salmonella* infection among animals housed together during the experiment. Such transmission would result in super-infections, re-infections (if the pig actually recovered before it was infected again) or passive shedding of the ingested *Salmonella*, all of which would cause underestimation of the duration of non-shedding states and overestimation of the duration of shedding states. We cannot rule out these possibilities. However, the first two possibilities seem unlikely because some pigs developed antibodies to *Salmonella* which presumably have had a protective effect. Moreover, experimental exposure of naïve pigs to shedding pigs has shown that the transmission rates between pigs are low [36]. Similarly, passive shedding does not seem likely because it would require ingestion of a considerable amount of fecal material that would survive a pig's digestive tract and be shed in high enough amounts to be detected. Finally, it is not obvious how any of the three possibilities could explain the observed association between a pig's transition through states of fecal shedding post-exposure and the challenge serotype and dose.

The performed analysis of immune responses indicated that pigs infected with the high dose of *S.* Typhimurium or *S.* Derby seroconverted 8 to 11 days faster than those infected with the low dose of these serotypes. Our analyses also indicated that these estimates strongly depend on the chosen manufacturer or custom derived cut-offs of the seroconversion level. Specifically, using manufacturer as opposed to custom cut-offs resulted in a longer estimated time to seroconversion for *S.* Typhimurium and *S.* Derby: approximately 5 days (or 30%) in pigs challenged with the low dose and approximately 3 days (or 50%) in pigs challenged with the high dose. Seroconversion to *S.* Cubana and *S.* Yoruba was approximately 4 and 14 times slower, respectively, than seroconversion to *S.* Typhimurium and *S.* Derby in pigs infected with the high dose. When manufacturer cut-offs were used, seroconversion to *S.* Yoruba was 7 times slower than that estimated for *S.* Typhimurium and *S.* Derby. That may be because *S.* Typhimurium and *S.* Derby are more invasive than *S.* Cubana and *S.* Yoruba and therefore invade the intestinal epithelium to a greater extent and induce antibody secretions more rapidly, in higher amounts, and over longer time periods than the feed-associated serotypes. Because pigs infected with the low dose or with *S.* Cubana or *S.* Yoruba need more time to seroconvert, more time would pass before the infection could be detected in the infected herd. While the custom cut-off has not been validated and so its validity is unknown, it is reasonable to speculate that for the tested serotypes it has a very good sensitivity but questionable specificity (except for the custom cut-off for *S.* Yoruba which was set so to achieve better specificity) and so may have a considerable number of false positive classifications. As a consequence, the time to seroconversion might be underestimated for *S.* Typhimurium, *S.* Derby, and *S.* Cubana in the model which used the custom cut-offs. On the other hand, it is reasonable to speculate that the manufacturer-recommended cut-offs might have a superior specificity at the cost of a reduced sensitivity to reduce the possibility of false positive classifications. Under these circumstances the model might have overestimated the time to seroconversion. The custom cut-off was useful in our setting to detect immune responses in pigs that were known to be exposed. It would be less useful in the field when the aim is to detect infected animals and the true status of exposure is unknown. Furthermore, test specificity is particularly important in societies where *Salmonella* control policy imposes mandatory restrictions and/or interventions in infected herds (such as movement restrictions of positive herds and euthanasia of positive pigs).

There are limited experimental studies reporting *Salmonella* fecal shedding and immune responses in pigs over a period of time based on frequent enough sample collection and testing. Nevertheless, the available information [11], [24], [25] compares favorably with the findings in this study. Previous studies of fecal shedding and immune response dynamics have used survival analysis and logistic regression models to find risk factors associated with these processes [37]–[41]. Only one previous study investigated the effect of time-varying covariates on *L. monocytogenes* fecal shedding using a Markov Chain modeling approach [35]. Our study demonstrated that multistate Markov models are a useful statistical tool for the analysis of longitudinal fecal shedding and immune response data. The regression analysis, which included a search for the most parsimonious representation of a covariate's effect on shedding and immune response, assured that the models are not overfitted with redundant parameters and also provided meaningful information about the effects the covariate has on the processes [13]. The relatively short duration of the study period and the small number of pigs per each covariate group in the pig dataset [9], [10] hampered the interpretation of some of the study results (i.e., estimates of sojourn times longer than the study period and very large standard errors). Nevertheless, using the multistate Markov Chain modeling approach, we were able to quantify and formally test previous reports of the effect of challenge serotype and dose on *Salmonella* fecal shedding and immune response and identify interesting new insights in the relationship between shedding episodes and total length of infection and shedding.

Our study could be used to suggest new avenues for research. For example, we emphasize the need to perform long enough longitudinal studies of fecal shedding and immune response to quantify the duration of fecal shedding states and immunity, particularly for high infection doses and classical pig serotypes. Since stress has been speculated to affect fecal shedding in pigs [42] and has been shown to affect the dynamics of fecal shedding of *L. monocytogenes* in cattle [35], it would be of interest to include the effect of stress in the future analysis of the dynamics of pigs' fecal shedding. In parallel, one could use some of the results of this study to build a mathematical infectious diseases model and to assess how challenge dose and serotype affect transmission of the pathogen within a population of pigs. Moreover, the results from this study may be applied in further models on within-herd transmission of *Salmonella* and the effects of different intervention and/or prevention measures.

In conclusion, the results of multistate modeling described in this study provided quantification and formal assessment of the previously described natural course of fecal shedding and immune responses post *Salmonella* challenge based on which new insights were possible. These results contributed to an understanding of the epidemiology of salmonellosis in pigs, demonstrating that the *Salmonella* serotype and the dose of exposure have profound effects on the pattern and duration of fecal shedding and host immune response. Such understanding may help improve the screening of different *Salmonella* serotypes in the pig reservoir, thereby decreasing the risk of human infection.

## References

[1] World Health Organization (WHO) (2005). Drug-resistant *Salmonella*. Fact sheet N°139.. http://www.who.int/mediacentre/factsheets/fs139/en/print.html.

[2] Centers for Disease Control and Prevention (CDC) (2006). Reported Foodborne Disease Outbreaks and Illnesses by Etiology and Food Commodities, United States.. http://www.cdc.gov/outbreaknet/surveillance_data.html.

[3] Doyle ME, Kaspar C, Archer J, Klos R (2009). White Paper on Human Illness Caused by *Salmonella* from all Food and Non-Food Vectors. FRI Briefings.. http://fri.wisc.edu/docs/pdf/FRI_Brief_Salmonella_Human_Illness_6_09.pdf.

[4] Hendriksen SW, Orsel K, Wagenaar JA, Miko A, van Duijkeren E (2004). Animal-to-human transmission of *Salmonella* Typhimurium DT104A variant.. Emerg Infect Dis.

[5] Fedorka-Cray PJ, Whipp SC, Isaacson RE, Nord N, Lager K (1994). Transmission of *Salmonella* Typhimurium to swine.. Vet Microbiol.

[6] Sanchez J, Dohoo IR, Christensen J, Rajic A (2007). Factors influencing the prevalence of *Salmonella* spp. in swine farms: a meta-analysis approach.. Prev Vet Med.

[7] Charles SD, Abraham AS, Trigo ET, Jones GE, Settje TL (2000). Reduced shedding and clinical signs of *Salmonella* Typhimurium in nursery pigs vaccinated with a *Salmonella* Choleraesuis vaccine.. Swine Health Prod.

[8] Gray JT, Stabel TJ, Fedorka-Cray PJ (1996). Effect of dose on the immune response and persistence of *Salmonella* choleraesuis infection in swine.. Am J Vet Res.

[9] Österberg J, Wallgren P (2008). Effects of a challenge dose of *Salmonella* Typhimurium or *Salmonella* Yoruba on the patterns of excretion and antibody responses of pigs.. Vet Rec.

[10] Österberg J, Lewerin Sternberg S, Wallgren P (2009). Patterns of excretion and antibody responses of pigs inoculated with *Salmonella* Derby and *Salmonella* Cubana.. Vet Rec.

[11] Nielsen B, Baggesen D, Bager F, Haugegaard J, Lind P (1995). The serological response to *Salmonella* serovars typhimurium and infantis in experimentally infected pigs. The time course followed with an indirect anti-LPS ELISA and bacteriologicalexaminations.. Vet Microbiol.

[12] van Winsen RL, van Nes A, Keuzenkamp D, Urlings HA, Lipman LJ (2001). Monitoring of transmission of *Salmonella enterica* serovars in pigs using bacteriological and serological detection methods.. Vet Microbiol 80(3),.

[13] Marshall G, Jones RH (1995). Multi-state Markov models and diabetic retinopathy.. Stat Med.

[14] Hougaard P (1999). Multi-state Model: A Review.. Lifetime Data Anal.

[15] Meira-Machado L, Cadarso-Suárez C, de Uña-Alvarez J (2007). tdc.msm: an R library for the analysis of multi-state survival data.. Comput Methods Programs Biomed.

[16] Eriksson E, Aspan A (2007). Comparison of culture, ELISA and PCR techniques for salmonella detection in faecal samples for cattle, pig and poultry.. BMC Vet Res.

[17] Jackson C (2010). Multi-state Markov and hidden Markov models in continuous time. R Documentation.. http://rss.acs.unt.edu/Rdoc/library/msm/html/msm.html.

[18] Kalbfleisch JD, Lawless JF (1985). The analysis of panel data under a Markov assumption.. Journal of the American Statistical Association.

[19] R Development Core Team (2004). R: A language and environment for statistical computing. R Foundation for Statistical Computing. Vienna, Austria. 3-900051-07-0.. http://www.R-project.org/.

[20] Jackson C (2007). Multi-state modelling with R: the msm package. Version 0.7.4. 1.. http://rss.acs.unt.edu/Rdoc/library/msm/doc/msm-manual.pdf.

[21] Kay R (1986). A Markov model for analysing cancer markers and disease states in survival studies.. Biometrics.

[22] Gentleman RC, Lawless JF, Lindsey JC, Yan P (1994). Multi-state Markov models for analysing incomplete disease history data with illustrations for HIV disease.. Stat Med.

[23] Gill J, King G (2004). What to do when your Hessian is not invertible.. Sociological Methods and Research.

[24] Scherer K, Szabo I, Rosler U, Appel B, Hensel A (2008). Time course of infection with *Salmonella* Typhimurium and its influence on fecal shedding, distribution in inner organs, and antibody response in fattening pigs.. J Food Protection.

[25] Jensen AN, Dalsgaard A, Stockmarr A, Nielsen EM, Baggesen DL (2006). Survival and Transmission of *Salmonella enterica* Serovar Typhimurium in an Outdoor Organic Pig Farming Environment.. Appl Environ Microbiol.

[26] Doyle PM Zhao T Meng J Zhao S 1997 Foodborne Pathogenic Bacteria. Escherichia coli O157:H7. In *Food Microbiology Fundamentals and Frontiers* Edited by Doyle MP, Beuchat L, and Montville TJ. ASM Press, Washington D.C 171 191

[27] Rocourt J Cossart P 1997 Foodborne Pathogenic Bacteria. *Listeria monocytogenes* In *Food Microbiology Fundamentals and Frontiers* Edited by Doyle MP, Beuchat L, Montville TJ. ASM Press, Washington D.C 337 352

[28] Molla B, Sterman A, Mathews J, Artuso-Ponte V, Abley M, at al (2010). *Salmonella enterica* in commercial swine feed and subsequent isolation of phenotypically and genotypically related strains in fecal samples.. Appl Environ Microbiol.

[29] Boqvist S, Hanson I, Nord Bjerselius U, Hamilton C, Wahlstrom H, at al (2003). *Salmonella* isolated from animals and feed production in Sweden between 1993 and 1997.. Acta Vet Scand.

[30] Malmqvist M, Jacobsson KG, Haggblom P, Cerenius F, Sjoland L (1995). *Salmonella* isolated from animals and feedstuffs in Sweden during 1988–1992.. Acta Vet Scand.

[31] Österberg J, Vagsholm I, Boqvist S, Sternberg Lewerin S (2006). Feed-borne outbreak of *Salmonella* Cubana in Swedish pig farms: risk factors and factors affecting the restriction period in infected farms.. Acta Vet Scand.

[32] Österberg J, Ekwall S, Nilsson I, Stampe S, Engvall A (2001). Eradication of *Salmonella* Yoruba in an integrated pig herd.. Berl Munch Tierarztl Woschenschr.

[33] Taormina PJ, Beuchat LR, Slutsker L (1999). Infections associated with eating seed sprouts: an international concern.. Emerg Infect Dis.

[34] Ivanek R, Snary EL, Cook AJC, Gröhn YT (2004). A mathematical model for the transmission *of Salmonella* Typhimurium within a grower-finisher pig herd in Great Britain.. J Food Prot.

[35] Ivanek R, Gröhn YT, Jui-Jung Ho A, Wiedmann M (2007). Markov chain approach to analyze the dynamics of pathogen fecal shedding–example of *Listeria monocytogenes* shedding in a herd of dairy cattle.. J Theor Biol.

[36] Österberg J, Sternberg Lewerin S, Wallgren P (2010). Direct and indirect transmission of four *Salmonella enterica* serotypes in pigs.. Acta Vet Scand.

[37] Bahnson PB, Fedorka-Cray PJ, Ladely SR, Mateus-Pinilla NE (2006). Herd-level risk factors for *Salmonella enterica* subsp. enterica in U.S. market pigs.. Prev Vet Med.

[38] García-Feliz C, Carvajal A, Collazos JA, Rubio P (2009). Herd-level risk factors for faecal shedding of *Salmonella enterica* in Spanish fattening pigs.. Prev Vet Med.

[39] Huston CL, Wittum TE, Love BC (2002). Persistent fecal *Salmonella* shedding in five dairy herds.. J Am Vet Med Assoc.

[40] Korsak N, Degeye JN, Etienne G, Beduin JM, China B, at al (2006). Use of a serological approach for prediction of *Salmonella* status in an integrated pig production system.. Int J Food Microbiol.

[41] Radke BR (2003). A demonstration of interval-censored survival analysis.. Prev Vet Med.

[42] Callaway TR, Morrow JL, Edrington TS, Genovese KJ, Dowd S (2006). Social stress increases fecal shedding of *Salmonella* typhimurium by early weaned piglets.. Curr Issues Intest Microbiol.

